# Construction of a Mass Spectrum Library Containing Predicted Electron Ionization Mass Spectra Prepared Using a Machine Learning Model and the Development of an Efficient Search Method

**DOI:** 10.5702/massspectrometry.A0120

**Published:** 2023-04-13

**Authors:** Ayumi Kubo, Azusa Kubota, Haruki Ishioka, Takuhiro Hizume, Masaaki Ubukata, Kenji Nagatomo, Takaya Satoh, Mitsuyoshi Yoshida, Fuminori Uematsu

**Affiliations:** 1 JEOL Ltd., 3–1–2 Musashino, Akishima, Tokyo 196–8558, Japan

**Keywords:** GC-MS, electron ionization, mass spectrum prediction, library search

## Abstract

Electron ionization (EI) mass spectrum library searching is usually performed to identify a compound in gas chromatography/mass spectrometry. However, compounds whose EI mass spectra are registered in the library are still limited compared to the popular compound databases. This means that there are compounds that cannot be identified by conventional library searching but also may result in false positives. In this report, we report on the development of a machine learning model, which was trained using chemical formulae and EI mass spectra, that can predict the EI mass spectrum from the chemical structure. It allowed us to create a predicted EI mass spectrum database with predicted EI mass spectra for 100 million compounds in PubChem. We also propose a method for improving library searching time and accuracy that includes an extensive mass spectrum library.

## 1. INTRODUCTION

Electron ionization (EI) is the most commonly used ionization method in gas chromatography/mass spectrometry (GC/MS).^1)^ Fragment ions, which are characteristic of the structures of compounds, are mainly observed in the EI mass spectra. Therefore, the identification of compounds involves searching a library that compares the observed EI mass spectra with those of standard compounds stored in the library.^2)^ NIST20, the most widely used EI mass spectral library, which stores pairs of structural formulas and measured mass spectra, contains 300,000 registered compounds. On the other hand, PubChem is a library of compounds that includes the molecular structures of more than 100 million compounds as of 2022.^3)^ Except for compounds registered in both PubChem and NIST 20, the EI mass spectra of 99% of the compounds in PubChem do not exist and therefore are not avilable. This means that these compounds will not only be unidentified by NIST library searching, but also may result in false-positives.^4)^

The availability of standard compounds limits the expansion of the measured EI mass spectral library. To solve this issue, several methods^5–9)^ have been reported for qualitative analyses that uses EI mass spectra that are developed *in-silico*. Bauer and Grimme proposed Quantum Chemical Electron Ionization Mass Spectrometry (QCEIMS),^5)^ a procedure that predicts EI mass spectra from molecular structures based on first-principle calculations. QCEIMS can calculate an EI mass spectrum with high accuracy, but minutes to hours are needed to create one mass spectrum. Allen *et al.* proposed competitive fragmentation modeling for EI (CFM-EI),^6)^ which is a machine learning model that predicts EI mass spectra from molecular structures. CFM-EI can create one EI mass spectrum in less than 10 min. Wei *et al.* proposed Neural Electron-Ionization Mass Spectromtery (NEIMS),^7)^ a machine learning model that creates EI mass spectra from molecular fingerprints. By converting structural formulas into fixed-length fingerprints in advance, NEIMS improved the speed of calculation of the EI mass spectrum to within one millisecond. Zhang *et al.* proposed a machine learning model that predicts mass spectra from molecular structures^8)^ using Graph Convolution Networks (GCN).^10)^ They solved the bit-collision problem of circular fingerprints in NEIMS.

In the actual analysis of complex mixtures, constructing a large-scale EI library that is made *in-silico* in advance would be expected to reduce searching time. In this report, we propose the development of the predicted EI library containing 100 million compounds based on the PubChem (pEI library) and an efficient search method. First, the machine learning model (pEI model) to convert the molecular structures to the predicted EI mass spectrum was created using GCNs similar to that reported in reference 8. The pEI model was trained using EI mass spectra and their structural formulas contained in NIST20. We then constructed a pEI library using molecular structural formulas in PubChem using the pEI model. However, the brute-force approach for searching the pEI library was found to take a long time and was less accurate. To improve the efficiency of the search method from the extensive pEI library, we limited the candidates used for comparing mass spectra by using the molecular formulas of the target compounds. The method used for the identification of the molecular formulas using EI and soft ionization methods such as field ionization (FI) and chemical ionization (CI) was reported previously^11)^ and was found to be valid for this step. In this method, the elemental compositions of molecular formulas are calculated using accurate masses obtained in FI or CI mass spectra. In addition, the candidate molecular formulas were narrowed down by the elemental compositions of the fragment ions observed in the EI mass spectrum. We report herein on the details of creating and evaluating the pEI model and applying the efficient search method to identify compounds that are not contained in the NIST20 library.

## 2. METHODS

### 2.1. Creation of the pEI model

**Figure 1:**
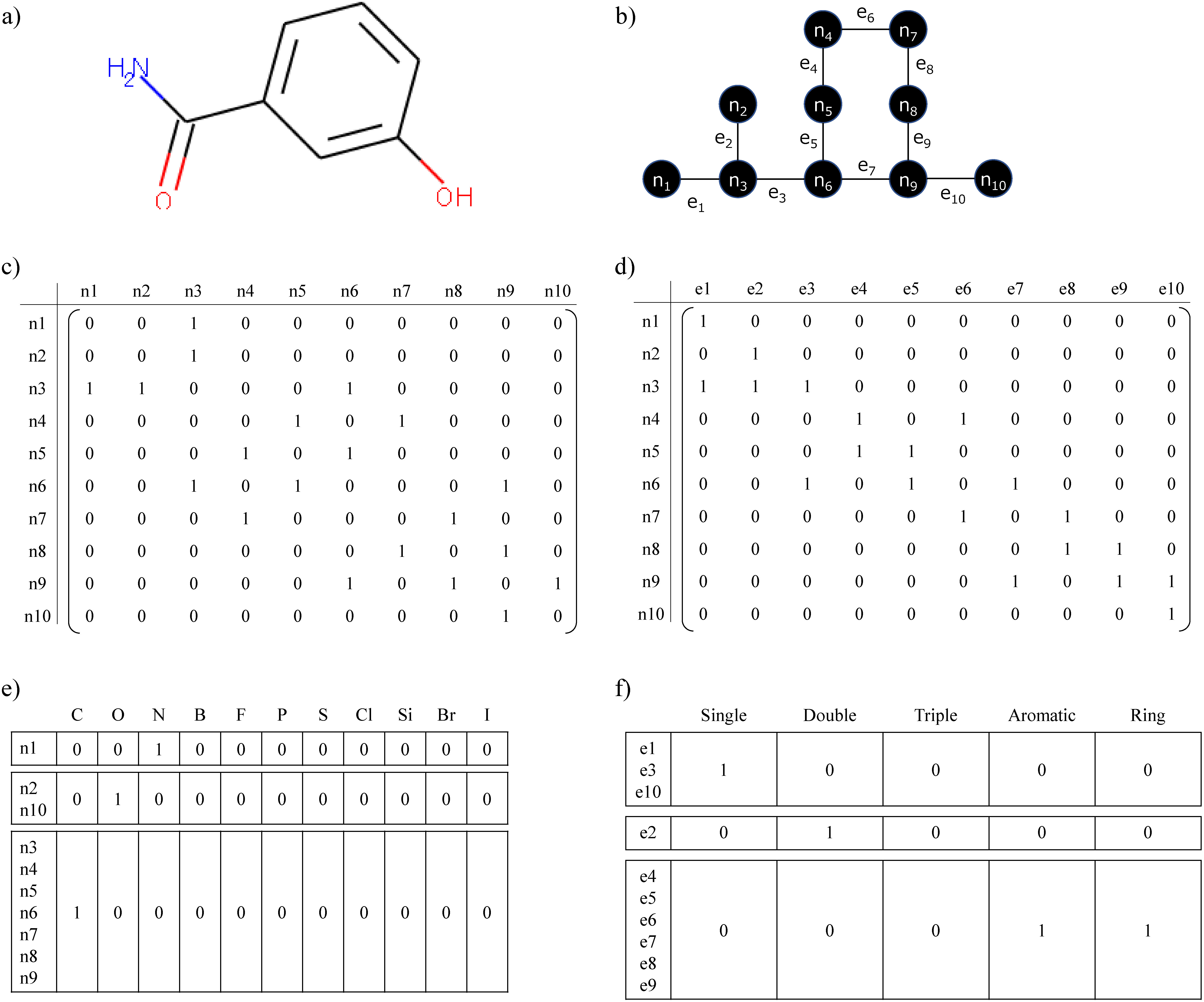
Fig. 1. Conversion of the chemical structure of 3-hydroxybenzamide to related graph data. The chemical structure (a) was converted to graph data (b). The relation of the node and edge was expressed in the adjacency matrix (c) and the incidence matrix (d). The nodes and edges also have the feature vectors (e) and (f), respectively.

The molecular structural formula is converted into graph data consisting of nodes and edges that connect them before being input into the GCN. The conversion was performed using RDKit^12)^ and DGL-LifeSci.^13)^ Figure 1 shows the strategy used to convert the structure of 3-hydroxybenzamide to graph data. Each atom and bond were treated as a node and an edge, respectively. The graph data for 3-hydroxybenzamide shown in Fig. 1b consisted of ten nodes, n1 to n10, connected by ten edges, e1 to e10. This structure was represented by the adjacency matrix shown in Fig. 1c and the incidence matrix in Fig. 1d. For example, node n1 is connected to node n3 by edge e1, so the value at row 1 of column 3 will be 1 in the adjacency matrix, and the value at row 1 of columns 1 and 3 will be 1 in the incidence matrix. Figure 1e shows the feature vectors of the nodes. Each node had a feature vector corresponding to elements that were limited to C/O/N/B/F/P/S/Cl/Si/Br/I which are observed in general GC–MS measurements. RDKit does not treat hydrogen atoms as nodes in graph data. For example, since node n3 is C, it has a featured vector (C, O, N, B, F, P, S, Cl, Si, Br, I)=(1, 0, 0, 0, 0, 0, 0, 0, 0, 0, 0). Figure 1f shows the feature vectors of the edges. Each edge had a feature vector corresponding to the type of bond. For example, edge e1 is a single bond, so it has the vector (single bond, double bond, double bond, aromatic bond, ring bond)=(1, 0, 0, 0, 0). In this method, the geometric isomers were not distinguished in the graph data because their observed EI mass spectra became similar.

**Figure 2:**
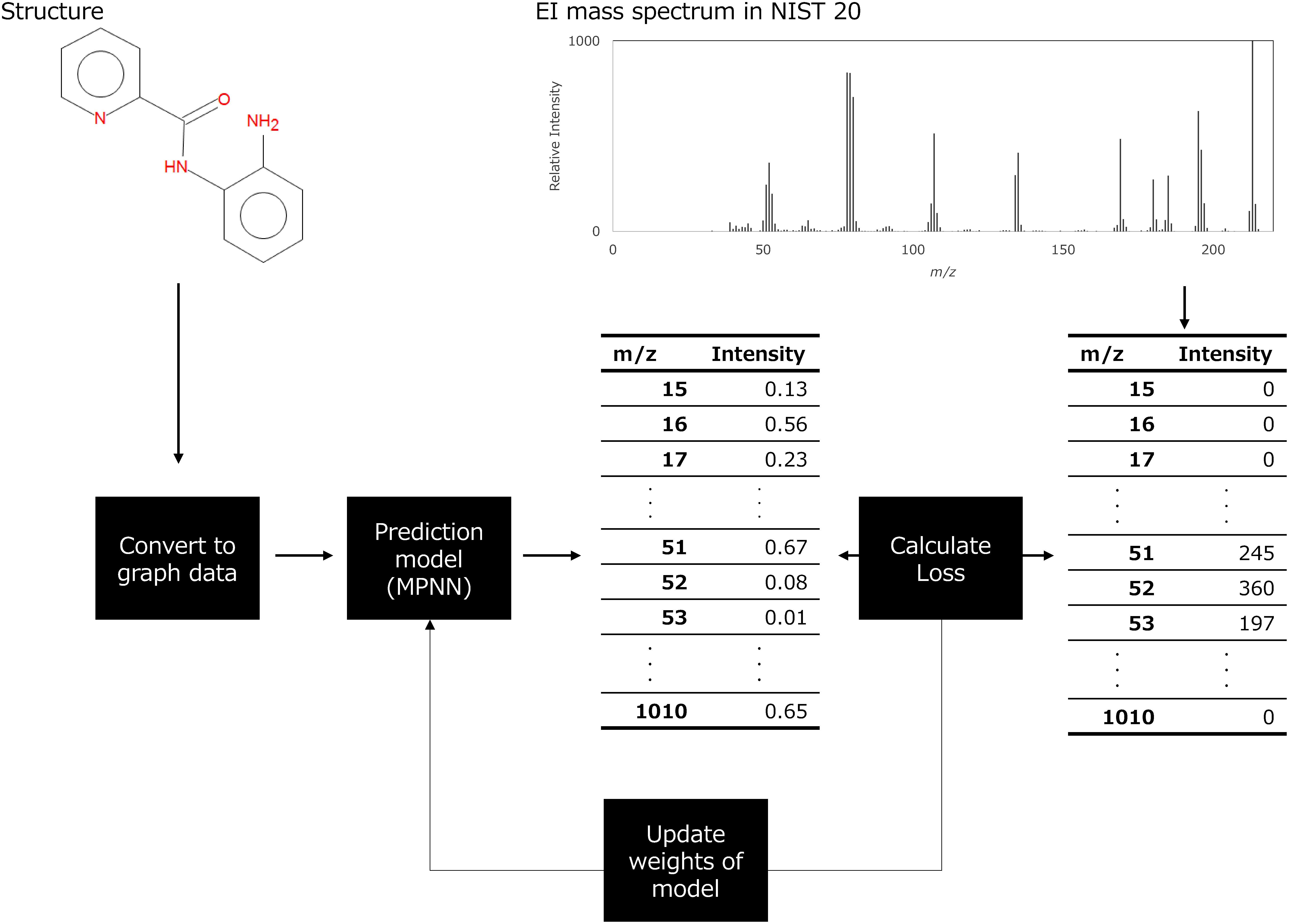
Fig. 2. The overview for training the message passing neural network (MPNN). The graph data were converted into EI mass spectra treated as a 996-dimensional vector corresponding to the ion intensities of *m*/*z* 15–1010. The MPNN was improved by using the 271,672 pairs of molecular structures and EI mass spectra stored in the mainlib of the NIST 20.

For training, we prepared a Python environment on a PC equipped with a GPU (NVIDIA GeForce RTX 3090) and installed PyTorch.^14)^ The overview for predicting the EI mass spectra from the graph data is shown in Fig. 2. A message passing the neural network (MPNN),^15)^ a type of GCN, was used to aggregate the graph data and to output the predicted EI mass spectrum. The MPNN was adopted because it can spatially recognize structural features. The output EI mass spectra were treated as a 996-dimensional vector corresponding to the ion intensities of *m*/*z* 15–1010. The main spectral library (mainlib) of NIST 20 was used for the training and evaluation of the model. About 90% of the mainlib, the 271,672 pairs of molecular structures and EI mass spectra (training dataset) were used for the training. Of the remaining data in mainlib, 10,000 pairs of molecular structures and EI mass spectra (validation dataset) were used for validation and 20,000 pairs (test dataset) were used for the test. Initially, the weights (coefficients) of the pEI model were set by random numbers, so the output EI mass spectrum was unrealistic. The loss function between the output EI mass spectra and the NIST20 mass spectra was calculated using Eq. (1) on the training dataset. Here, *m_i_* is an integer *m*/*z* value ranging from 15 to 1010, *A_i_* is the intensity of *m_i_* in the NIST EI mass spectrum, and *P_i_* is the intensity of *m_i_* in the output EI mass spectrum. 
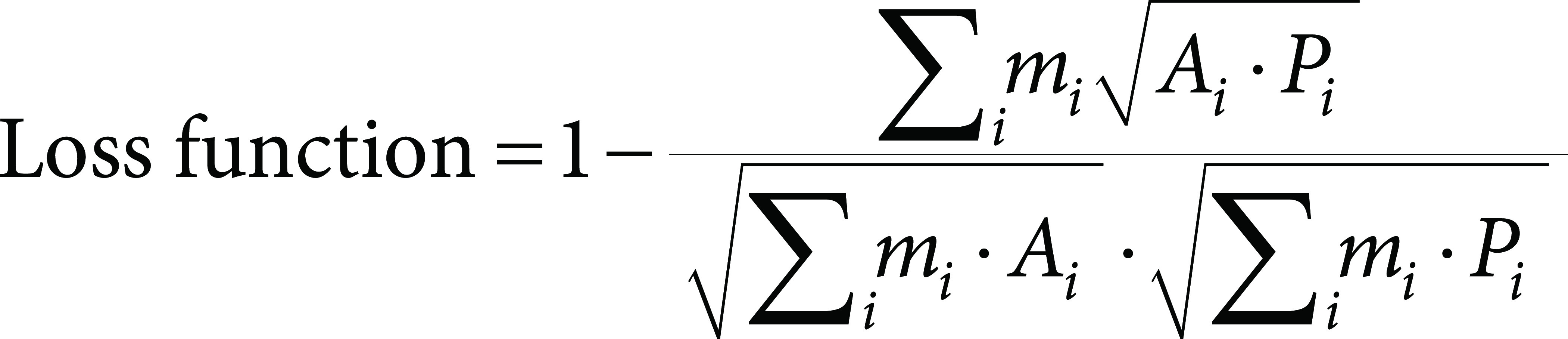
(1)

The weights of the pEI model were repeatedly updated (each repetition is referred to as an epoch) to reduce the loss function. At the end of each epoch, the loss function was also calculated on the validation dataset to avoid overfitting. The pEI model was considered to be improved if the loss function on the validation dataset decreased. The value of the loss function stopped decreasing after approximately 200 epochs, we therefore concluded that the training of the pEI model had progressed sufficiently. The weight of the pEI model was adopted when the loss function was minimized.

### 2.2. Construction of the pEI library

The pEI library was constructed based on PubChem, which contains 100 million recorded compounds as of February 2022. The predicted EI mass spectra were made by inputting the PubChem compound structure into the pEI model. The molecular formula and predicted EI mass spectra of each pair were recorded in the pEI library. However, three exceptions were not used for predicting EI mass spectra: (i) when multiple structures were combined and registered as one compound, (ii) in cases of compounds with a molecular weight over 1000, and (iii) compounds containing elements other than C/O/N/B/F/P/S/Cl/Si/Br/I, such as Na and Al. The total number of registered compounds in the pEI library was 96,912,831.

### 2.3. Searching the pEI library

The pEI library search was performed in two steps in order to reduce search time and improve accuracy. First, the candidates are extracted from the pEI library using the molecular formula identified for the measured compounds. The number of extracted candidates (the number of candidates with the same molecular formula but with different molecular structures) depends on the compounds, but it is typically less than 10,000. At this step, the number of candidates has been narrowed down from approximately 100 million to less than 1/10,000. Second, the cosine similarity between the predicted EI mass spectra of the extracted candidates and the measured EI mass spectra was calculated using Eq. (2). In this equation, *m_i_* is the integer *m*/*z* value ranging from 15 to 1010, *A_i_* is the intensity of the measured EI mass spectrum of *m_i_*, and *P_i_* is the intensity of the predicted EI mass spectrum of *m_i_*. 
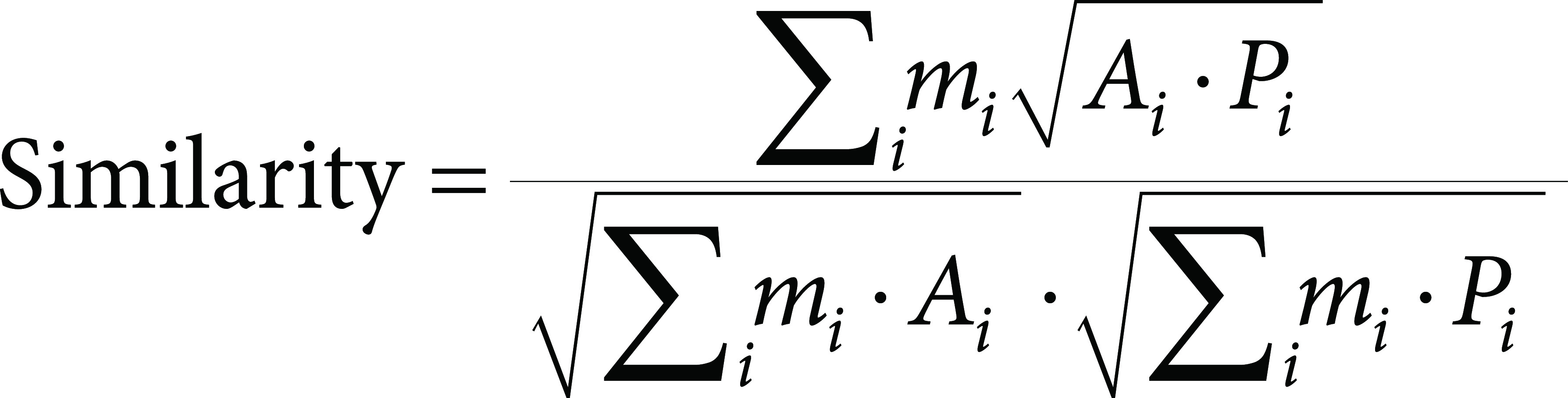
(2)

## 3. RESULTS AND DISCUSSION

**Figure 3:**
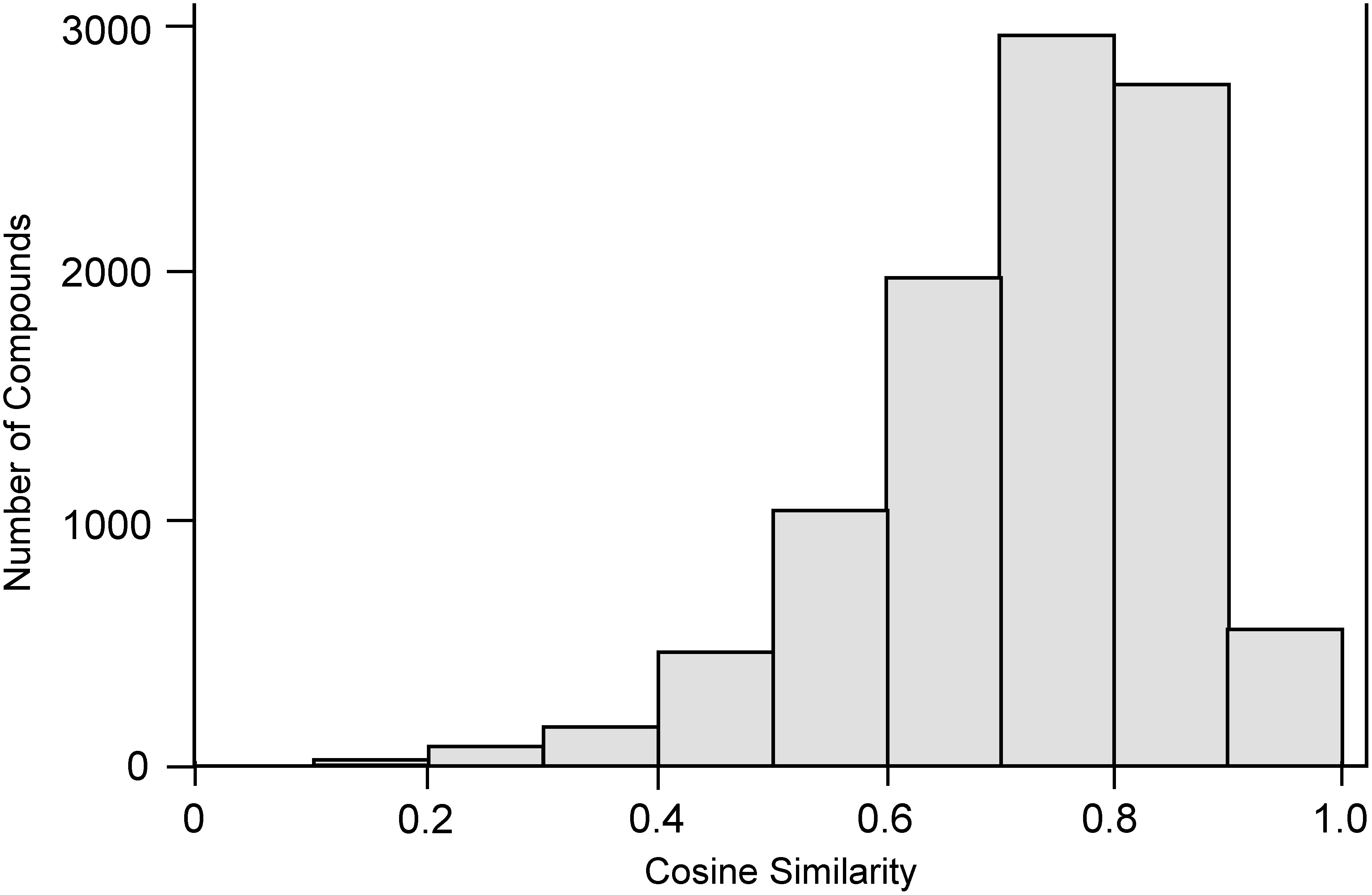
Fig. 3. Histogram of cosine similarities between observed and predicted EI mass spectra.

**Figure 4:**
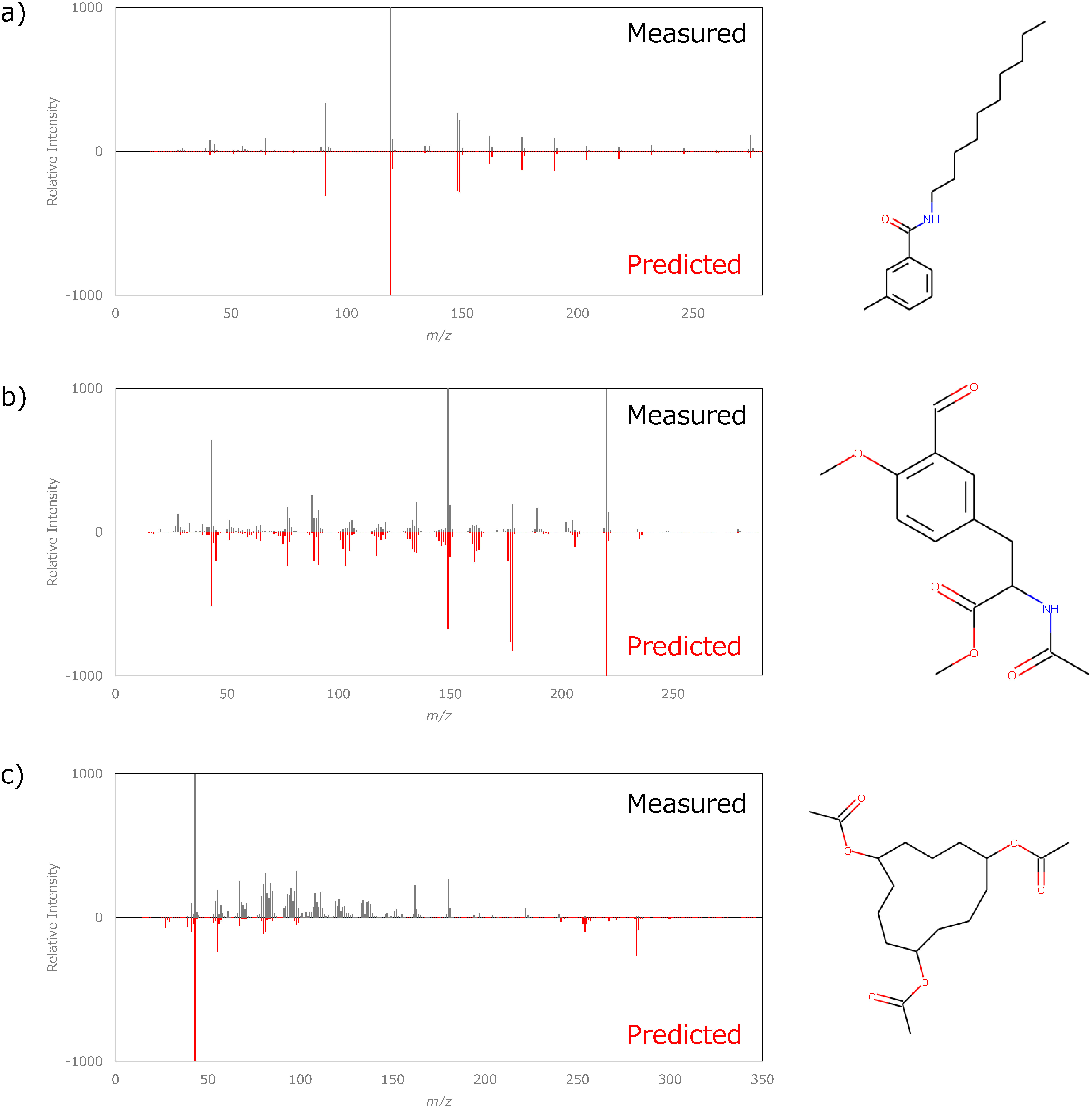
Fig. 4. Comparison of observed EI mass spectra and predicted EI mass spectra of benzamide, 3-methyl-*N*-decyl-[cosine similarity: 0.95] (a), *N*-acetyl-3-(3-formyl-4-methoxyphenyl)-D-alanine methyl ester [cosine similarity: 0.72] (b), and cyclododecane, 1,5,9-tris(acetoxy)-[cosine similarity: 0.34] (c).

We first evaluated the reconstruction error of the EI mass spectra prediction model using the test dataset. The cosine similarities between their observed EI mass spectra and predicted EI mass spectra were calculated to assess the reconstruction error. Figure 3 shows the calculated cosine similarity distribution: over 90% of the compounds had a cosine similarity of 0.40 or higher, and the overall mean average was 0.72. In Fig. 4, the observed EI mass spectra in NIST20 and the predicted EI mass spectra were compared for benzamide, 3-methyl-*N*-decyl-[cosine similarity 0.95] (a), *N*-acetyl-3-(3-formyl-4-methoxyphenyl)-D-alanine methyl ester [cosine similarity 0.72] (b), and cyclododecane, 1,5,9-tris(acetoxy)-[cosine similarity 0.34] (c). Benzamide, 3-methyl-*N*-decyl-, showed a nearly perfectly reproduced mass spectra even with weak mass peaks. This high similarity can be attributed to the fact that the compound consists only of benzene rings, alkyl chains, and amide groups that are all commonly seen in the compounds in NIST20. The *N*-acetyl-3-(3-formyl-4-methoxyphenyl)-D-alanine methyl ester showed good reproducibility for relatively high-intensity peaks. However, this compound has a rather complex structure, with a benzene ring with multiple side chains, so the reproducibilities of the minor peaks were relatively low. Cyclododecane, 1,5,9-tris(acetoxy)-, showed a lower reproducibility and only the most intense peak at *m*/*z* 43 could be reproduced. This compound contains a 12-membered ring that is rarely seen in NIST20. The poor prediction for this compound may be due to the lack of training data for compounds with 12-membered rings.

Second, the accuracy of the pEI library search method was evaluated using the test dataset. This evaluation was limited to the 14,581 compounds that had 100 or more candidates and the same molecular formula in the pEI library. We checked the rank for the correct structure using cosine similarity (Table 1). The correct molecular structure ranked in the top 10% for 93% of the compounds, and in the top 1% for 73% of the compounds. Here, being within the top 1% means that if there were 1000 extracted candidates, the correct molecular structure would be in the top 10. PubChem contains compounds with very similar structures, so the accuracy of a pEI library search method will be fairly high. Tables S1 to S5 show the correct and the top molecular structures for ten compounds in each of the five ranking categories, *i.e.*, the top, the top 1%, the top 1 to 5%, the top 5 to 10%, and greater than 10%. The tables also show comparisons of the measured EI mass spectra and EI mass spectra predicted from the correct molecular structures. Additionally, the correct molecular structure ranked at the top for 22% of the compounds, which is higher than the result of CFM-EI of 20% or less.^6)^

**Table 1:** Table 1. Accuracy of the pEI library search method, as evaluated by checking the rank of the correct structure using cosine similarity.

	Top	Within the top 1%	Within the top 5%	Within the top 10%
Number of compounds	3215 (22%)	10618 (73%)	12934 (89%)	13594 (93%)

Lastly, we evaluated the pEI library search method using the measured EI mass spectra of six compounds that have not been registered in NIST20: cafenstrole (CAS: 125306-83-4), MCPA-triethyl (CAS: 25319-90-8), propaphos (CAS: 7292-16-2), CNP-amino (CAS: 26306-61-6), butamifos oxon (CAS: 56362-05-1), and isoxadifen-ethyl (CAS: 163520-33-0). The EI mass spectra of the six compounds were obtained by gas chromatograph-mass spectrometer (JEOL Ltd.). The molecular formulas are shown in the second column of Table 2. The cosine similarity of each compound is shown in the third column of Table 2. The rank for each compound in the fourth column indicates the number of compounds with the same molecular formula (listed in parentheses) and the rank of similarity among them. The top ten structural formulas with the highest degree of similarity are also listed in the supporting information. Three out of six compounds resulted in the highest degree of similarity. In the results of the pEI library search method, the lowest-ranking compound was isoxadifen-ethyl, which ranked 22nd among 5348 candidates; however, the correct structure was in the top 1%, demonstrating the effectiveness of this method. The top ten structural formulas with the highest degree of similarity are listed in Table S6. In comparing the actual molecular structures of cafenstrole, CNP-amino, and isoxadifen-ethyl with the high-ranked molecular structures, reasonably good agreement was found, such as the number of benzene rings and the presence or absence of heterocycles or side chains. Overall, the prediction appears to be largely accurate. Additionally, a brute-force search of the entire pEI library was performed. The rank for each compound is listed in the fifth column. Compared with the results for the brute-force search and the pEI library search, the correct structures were ranked higher in the latter case. For example, MCPA-thioethyl was improved from the 232nd to the top position. Futhermore, the pEI library search requires 4 s per compound, while the brute-force search requires about two hours per compound. Therefore, our pEI model and pEI search method appears to be useful for structural analysis.

**Table 2:** Table 2. Evaluation of the accuracy of the pEI library search method for six compounds that were not registered in NIST 20.

Compound name	Formula	Similarity	Rank: pEI library search	Rank: brute-force search
Cafenstrole	C_16_H_22_N_4_O_3_S	0.741	3 (2933)	229
MCPA-thioethyl	C_11_H_13_ClO_2_S	0.735	1 (729)	232
Propaphos	C_13_H_21_O_4_PS	0.802	1 (27)	1
CNP-amino	C_12_H_8_Cl_3_NO	0.710	14 (618)	1254
Butamifos oxon	C_13_H_21_N_2_O_5_P	0.675	1 (56)	12822
Isoxadifen-ethyl	C_18_H_17_NO_3_	0.586	22 (5348)	42199

## 4. CONCLUSIONS

The number of mass spectra in the popular EI library is still limited compared to the compound database. In this report, we proposed a method for creating a predicted EI mass spectrum by means of a machine learning model and a method for searching the 100 million compound library. This demonstrated the extensibility of the structural analysis of unknown compounds found in GC/MS analysis, which is not listed in the conventional EI mass spectrum libraries.

## Notes

Mass Spectrom (Tokyo) 2023; 12(1): A0120
