# Supplementary material for: Construction of a Mass Spectrum Library Containing Predicted Electron Ionization Mass Spectra Prepared Using a Machine Learning Model and the Development of an Efficient Search Method
Source: Mass Spectrom (Tokyo). 2023 Apr 13;12(1):A0120. doi: 10.5702/massspectrometry.A0120 (PMC10209659; doi:10.5702/massspectrometry.A0120)
Supplement: Supplementary Data [file massspectrometry-12-1-A0120_s001.pdf]

Supporting Information

Construction of a mass spectrum library containing predicted electron ionization mass spectra made by machine learning model and development of an efficient search method

Ayumi Kubo[1*], Azusa Kubota[1], Haruki Ishioka[1], Takuhiro Hizume[1], Masaaki Ubukata[1], Kenji Nagatomo[1], Takaya Satoh[1], Mitsuyoshi Yoshida[1], Fuminori Uematsu[1]

[1] JEOL Ltd. Akishima, Tokyo, 196-8558, Japan

*Corresponding authors:

akubo@jeol.co.jp

Table S1 Accuracy evaluation result and comparison of measured EI mass spectra and EI mass spectra predicted from correct molecular structures for ten compounds with the top ranking of top.

Rank:<br>1 (2370)<br>Formula:<br>$C_{13}H_{21}NO_4S$<br>Similarity:<br>0.83	Correct/ Top<br>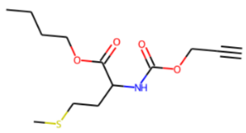	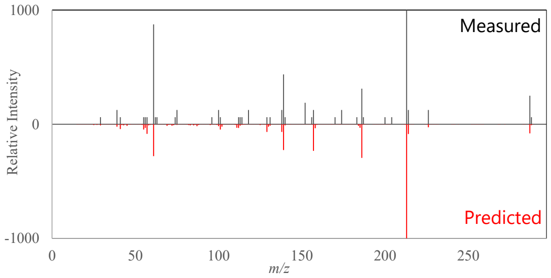
Rank:<br>1 (1185)<br>Formula:<br>$C_{19}H_{28}O_5$<br>Similarity:<br>0.88	Correct/ Top<br>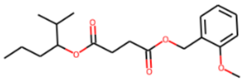	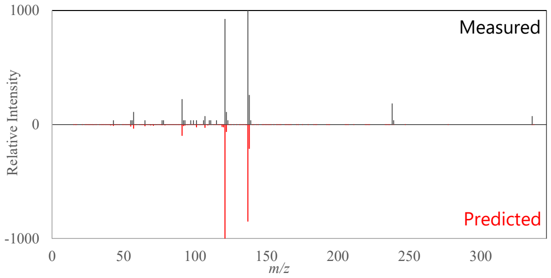
Rank:<br>1 (1514)<br>Formula:<br>$C_{14}H_{19}BrO_3$<br>Similarity:<br>0.72	Correct/ Top<br>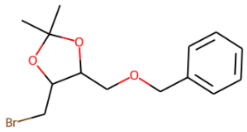	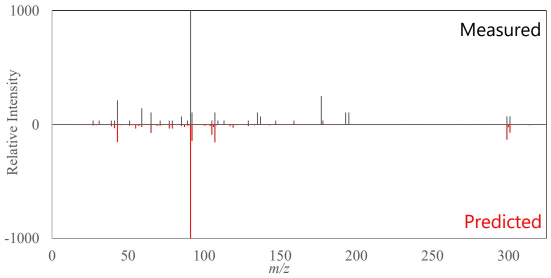
Rank:<br>1 (6655)<br>Formula:<br>$C_{14}H_{15}N_3O_3S$<br>Similarity:<br>0.81	Correct/ Top<br>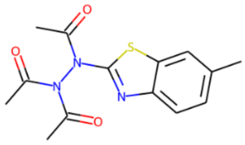	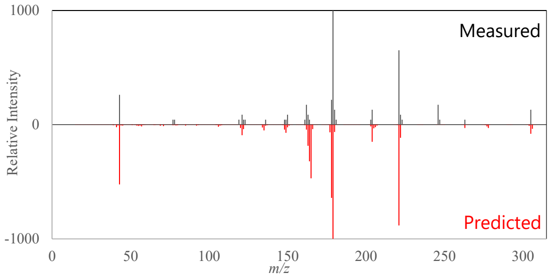
Rank:<br>1 (222)<br>Formula:<br>$C_{18}H_{31}NO_2S$<br>Similarity:<br>0.83	Correct/ Top<br>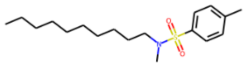	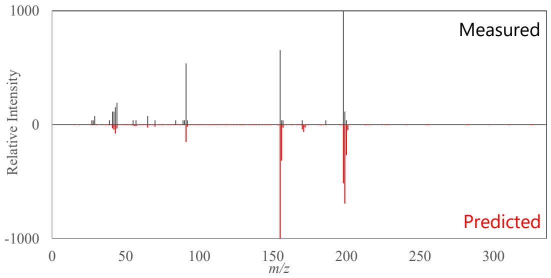

Table S1 continued

Rank:<br>1 (101)<br>Formula:<br>$C_{21}H_{29}NOSi$<br>Similarity:<br>0.75	Correct/ Top<br>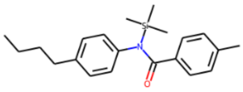 	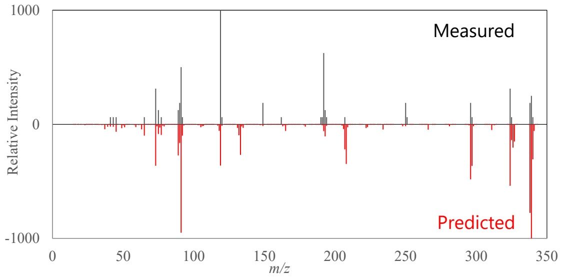
Rank:<br>1 (2209)<br>Formula:<br>$C_{13}H_{12}BrN_3O_2$<br>Similarity:<br>0.79	Correct/ Top<br>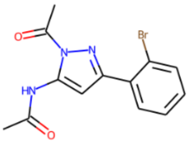 	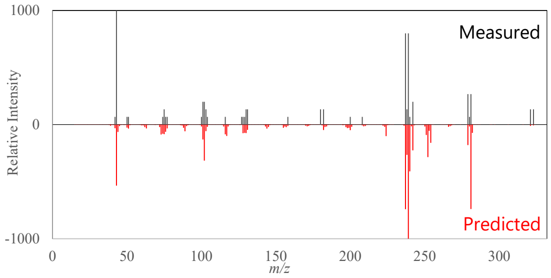
Rank:<br>1 (863)<br>Formula:<br>$C_{21}H_{18}ClNO_3$<br>Similarity:<br>0.66	Correct/ Top<br>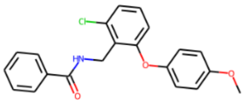 	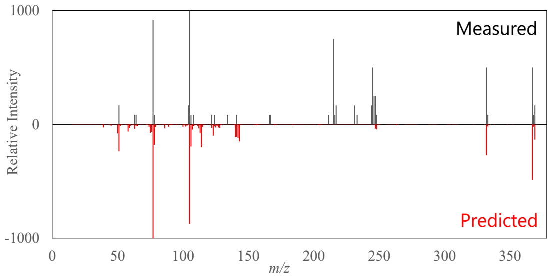
Rank:<br>1 (1911)<br>Formula:<br>$C_{16}H_{12}N_2O_4$<br>Similarity:<br>0.78	Correct/ Top<br>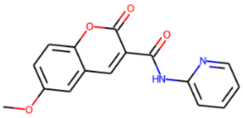 	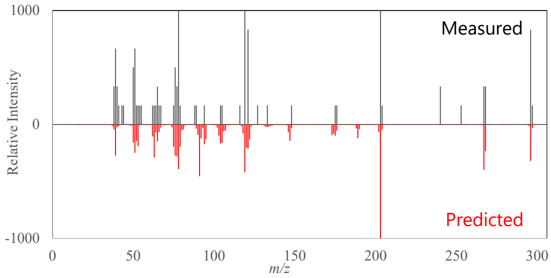
Rank:<br>1 (257)<br>Formula:<br>$C_{28}H_{43}NO_3$<br>Similarity:<br>0.58	Correct/ Top<br>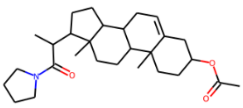 	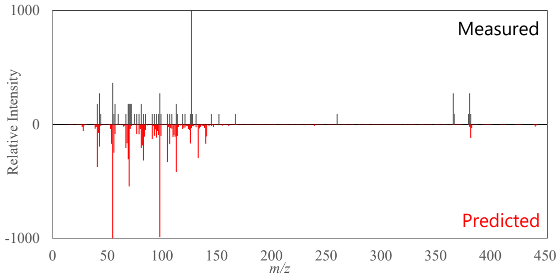

Table S2 Accuracy evaluation result and comparison of observed EI mass spectra and EI mass spectra predicted from the correct molecular structures for ten compounds with a ranking of 1% or less.

Rank:<br>2 (4567)<br>Formula:<br>$C_{18}H_{24}N_4O_3$<br>Similarity:<br>0.62	Correct<br>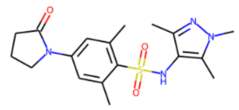<br>Top<br>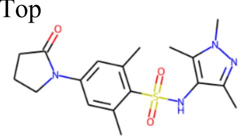	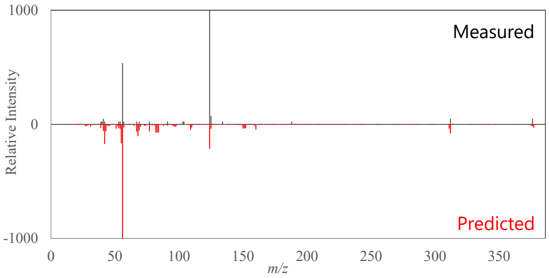
Rank:<br>5 (3204)<br>Formula:<br>$C_9H_{12}N_2O_2S$<br>Similarity:<br>0.73	Correct<br>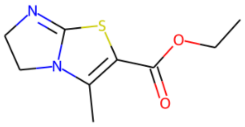<br>Top<br>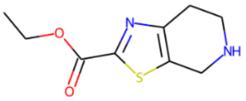	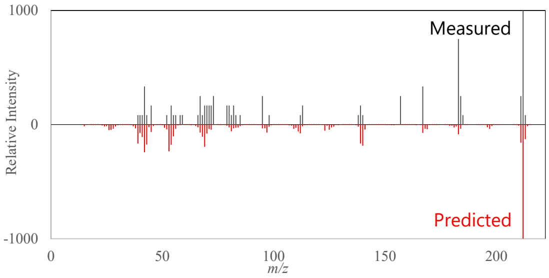
Rank:<br>3 (8796)<br>Formula:<br>$C_{12}H_{14}N_4O$<br>Similarity:<br>0.77	Correct<br>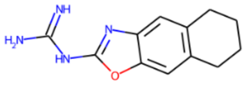<br>Top<br>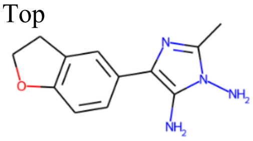	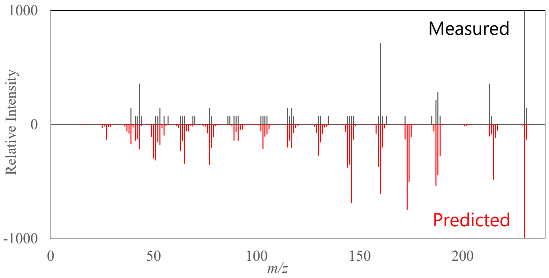
Rank:<br>6 (1961)<br>Formula:<br>$C_{11}H_{14}O_5$<br>Similarity:<br>0.73	Correct<br>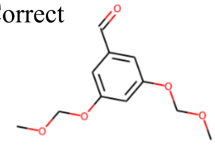<br>Top<br>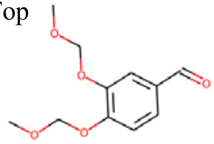	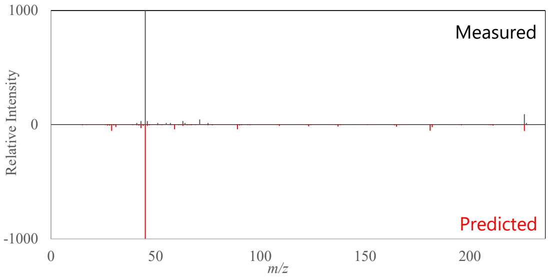
Rank:<br>3 (2462)<br>Formula:<br>$C_{16}H_{14}N_2O_2S$<br>Similarity:<br>0.70	Correct<br>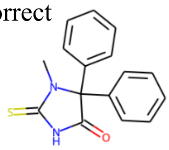<br>Top<br>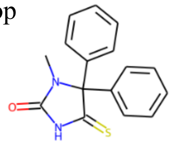	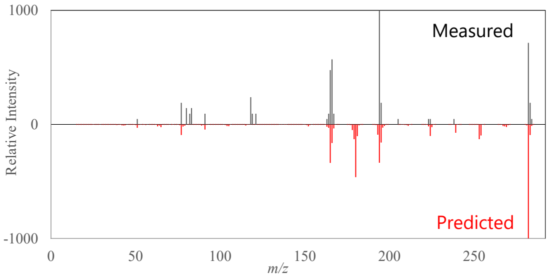

Table S2 continued

Rank: 11 (3062)<br>Formula: $C_{13}H_{16}N_4O_4$<br>Similarity: 0.74	Correct 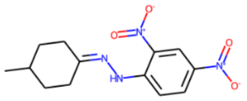	Top 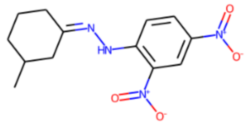	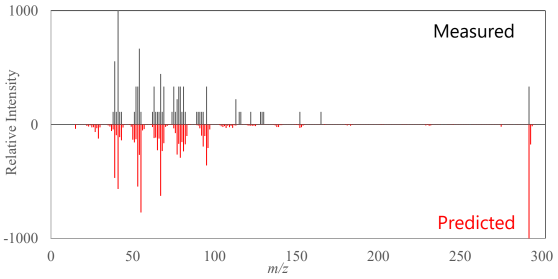
Rank: 3 (824)<br>Formula: $C_{23}H_{44}O_4$<br>Similarity: 0.89	Correct 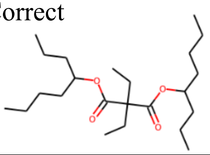	Top 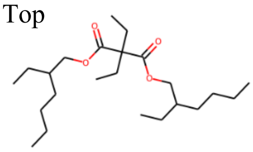	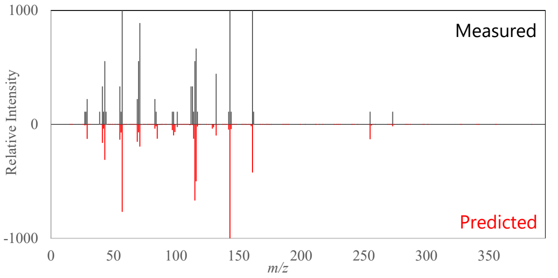
Rank: 3 (983)<br>Formula: $C_{18}H_{15}FN_2O_2S$<br>Similarity: 0.80	Correct 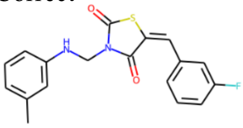	Top 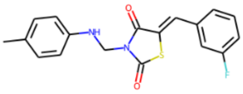	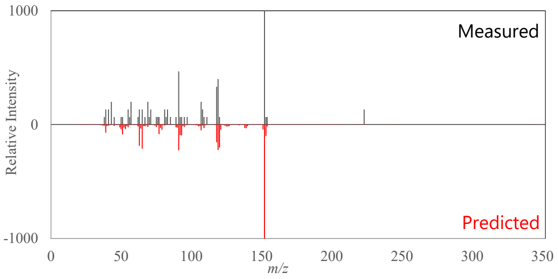
Rank: 3 (352)<br>Formula: $C_{21}H_{30}N_4O_6$<br>Similarity: 0.78	Correct 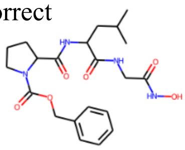	Top 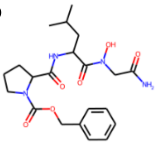	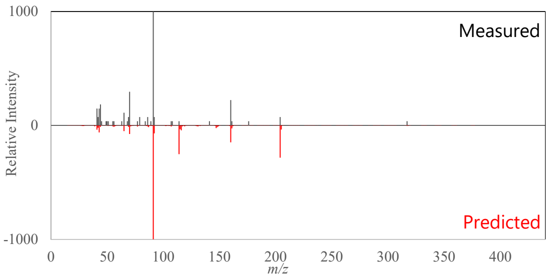
Rank: 2 (651)<br>Formula: $C_{27}H_{23}ClN_2O_4$<br>Similarity: 0.62	Correct 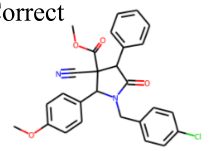	Top 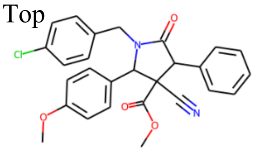	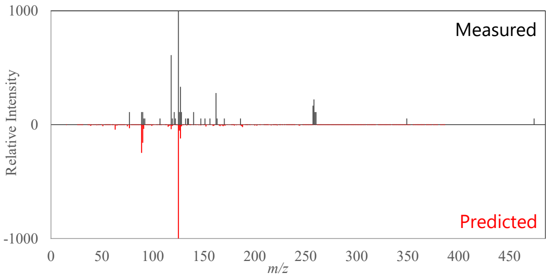

Measured

Table S3 Accuracy evaluation result and comparison of observed EI mass spectra and EI mass spectra predicted from the correct molecular structures for ten compounds with ranking of the top 1 to 5%.

Rank:<br>11 (535)<br>Formula:<br>$C_{14}H_{11}N$<br>Similarity:<br>0.91	Correct<br>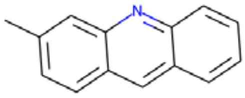<br>Top<br>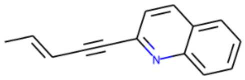	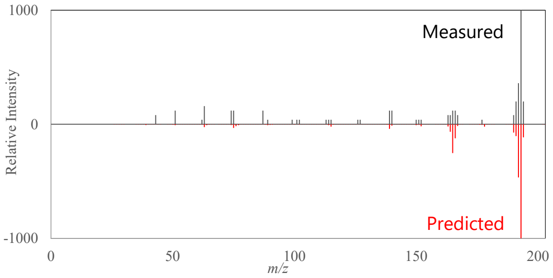
Rank:<br>145 (4283)<br>Formula:<br>$C_{16}H_{16}O_2$<br>Similarity:<br>0.51	Correct<br>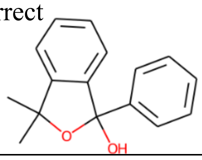<br>Top<br>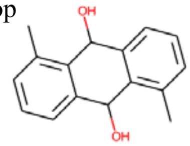	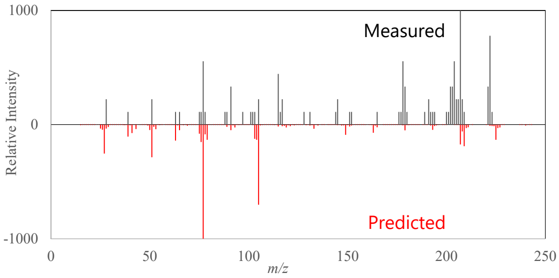
Rank:<br>3 (131)<br>Formula:<br>$C_{17}H_{14}Cl_2F_2N_2O_2$<br>Similarity:<br>0.71	Correct<br>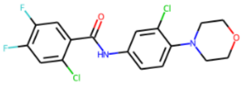<br>Top<br>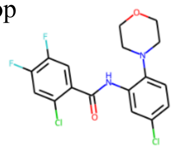	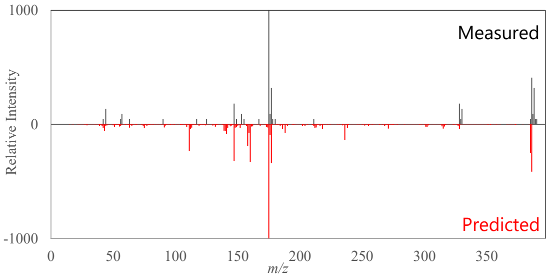
Rank:<br>4 (360)<br>Formula:<br>$C_{13}H_9N_5O_3$<br>Similarity:<br>0.72	Correct<br>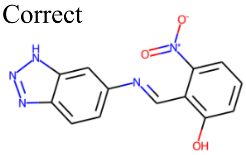<br>Top<br>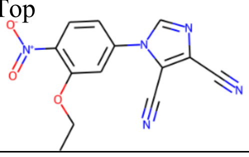	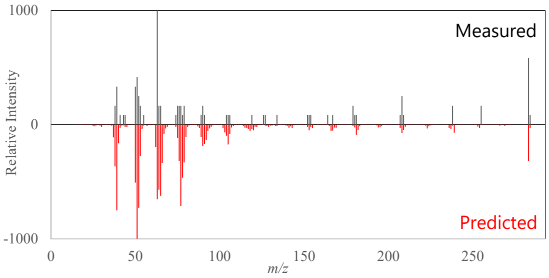
Rank:<br>2 (131)<br>Formula:<br>$C_9H_{15}O_4P$<br>Similarity:<br>0.72	Correct<br>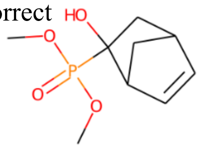<br>Top<br>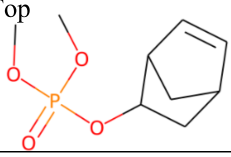	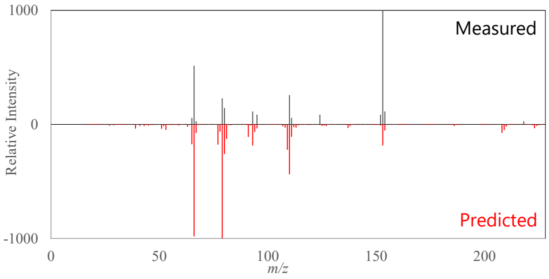

Table S3 continued

Rank: 85 (6887)<br>Formula: $C_{19}H_{18}N_2O_2$<br>Similarity: 0.65	Correct 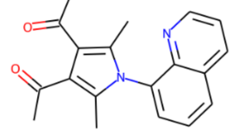<br>Top 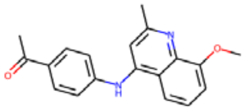	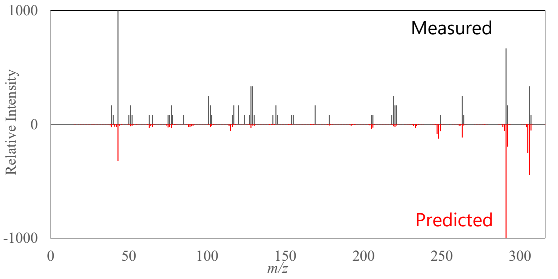
Rank: 4 (219)<br>Formula: $C_{17}H_{30}O_4Si$<br>Similarity: 0.67	Correct 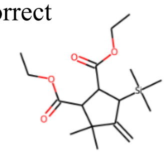<br>Top 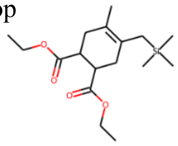	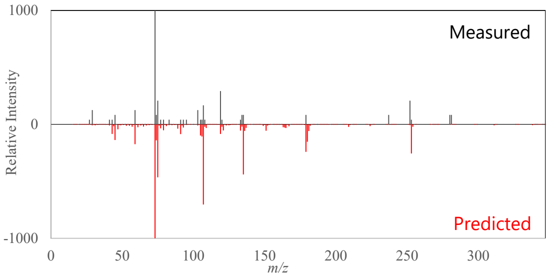
Rank: 16 (1374)<br>Formula: $C_{24}H_{20}N_2O_4S$<br>Similarity: 0.74	Correct 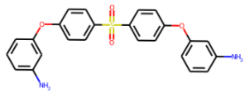<br>Top 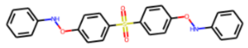	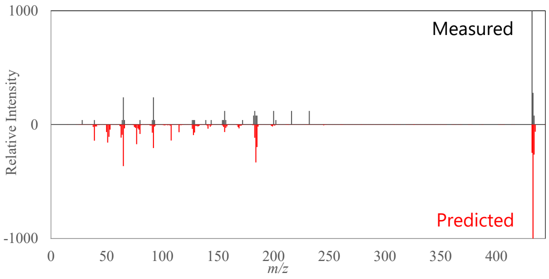
Rank: 7 (315)<br>Formula: $C_{23}H_{46}$<br>Similarity: 0.84	Correct 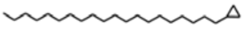<br>Top 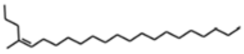	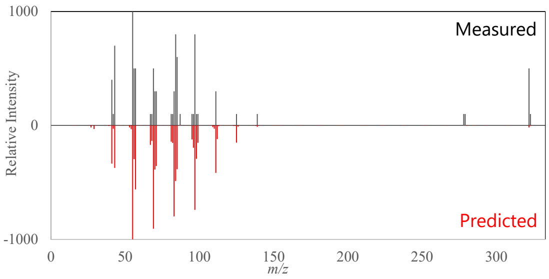
Rank: 34 (1283)<br>Formula: $C_{17}H_{16}ClN_5O$<br>Similarity: 0.67	Correct 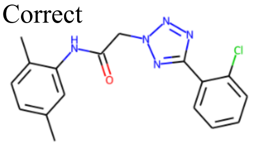<br>Top 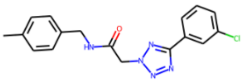	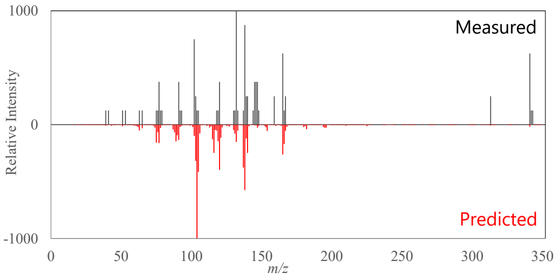

Table S4 Accuracy evaluation result and comparison of observed EI mass spectra and EI mass spectra predicted from the correct molecular structures for ten compounds with ranking of top 5 to 10%.

Rank: 52 (580)<br>Formula: $C_{15}H_{10}O_3$<br>Similarity: 0.79	Correct 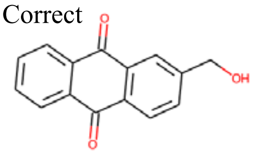<br>Top 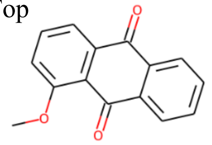	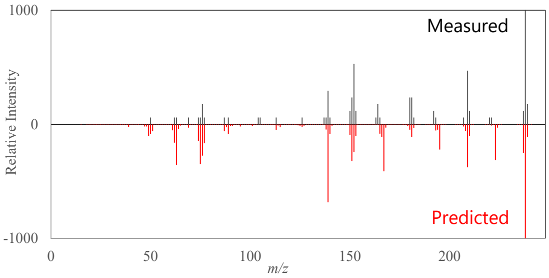
Rank: 313 (3934)<br>Formula: $C_{13}H_{12}N_2O$<br>Similarity: 0.79	Correct 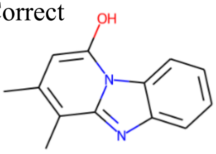<br>Top 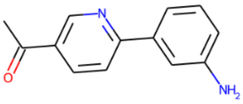	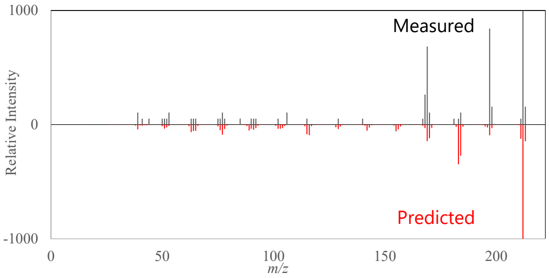
Rank: 52 (709)<br>Formula: $C_{10}H_8BrN_3O$<br>Similarity: 0.72	Correct 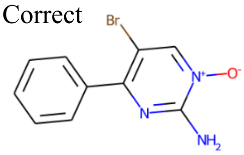<br>Top 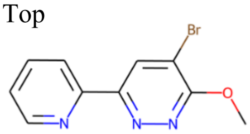	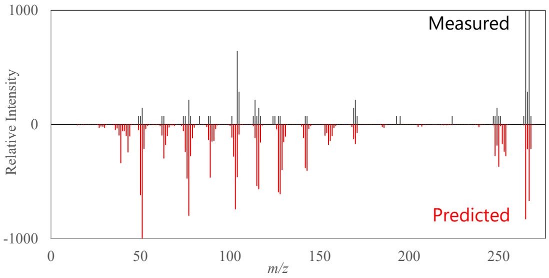
Rank: 8 (147)<br>Formula: $C_{23}H_{40}O_3Si$<br>Similarity: 0.63	Correct 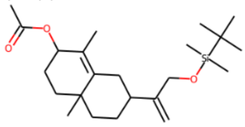<br>Top 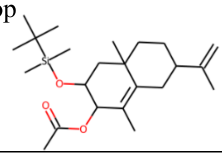	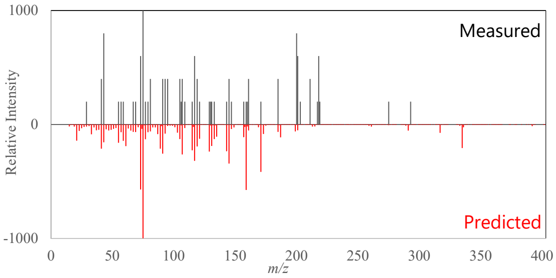
Rank: 387 (5613)<br>Formula: $C_{12}H_{14}N_2OS$<br>Similarity: 0.63	Correct 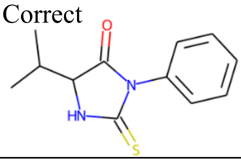<br>Top 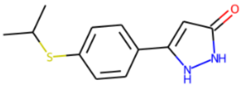	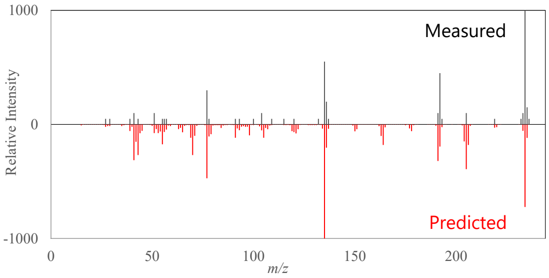

Table S4 continued

Rank: 65 (973)<br>Formula: $C_{13}H_{11}Cl_2NO$<br>Similarity: 0.61	Correct 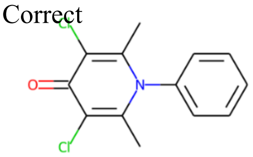<br>Top 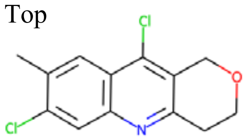	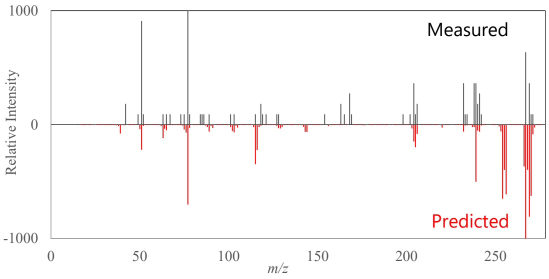
Rank: 12 (121)<br>Formula: $C_{20}H_{13}BrN_2O$<br>Similarity: 0.79	Correct 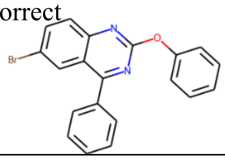<br>Top 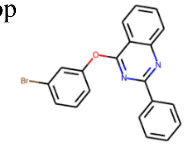	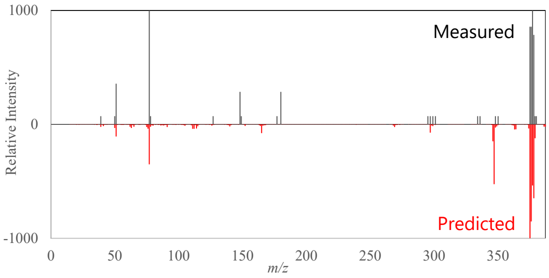
Rank: 509 (8122)<br>Formula: $C_{19}H_{22}N_2O_3S$<br>Similarity: 0.61	Correct 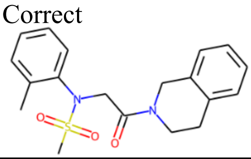<br>Top 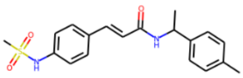	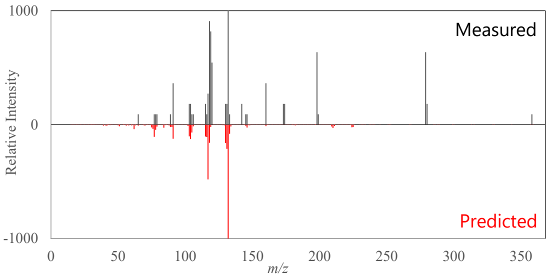
Rank: 140 (1717)<br>Formula: $C_{19}H_{30}O$<br>Similarity: 0.76	Correct 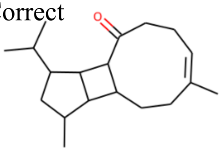<br>Top 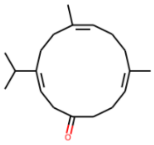	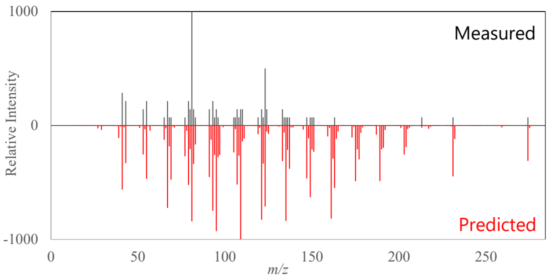
Rank: 12 (235)<br>Formula: $C_{27}H_{35}O$<br>Similarity: 0.56	Correct 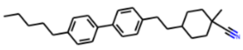<br>Top 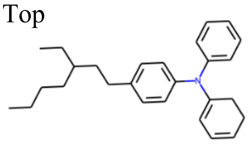	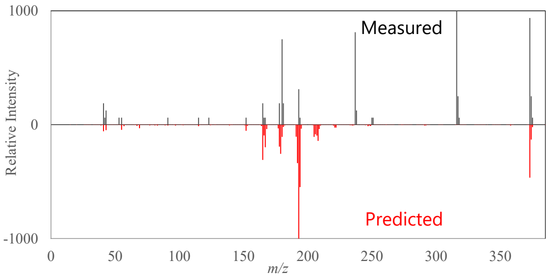

Table S5 Accuracy evaluation result and comparison of observed EI mass spectra and EI mass spectra predicted from the correct molecular structures for ten compounds in with a ranking greater than 10%.

Rank:<br>889 (4474)<br>Formula:<br>$C_{20}H_{18}N_2O_2$<br>Similarity:<br>0.46	Correct<br>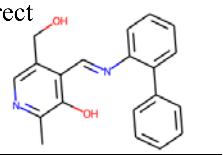<br>Top<br>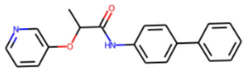	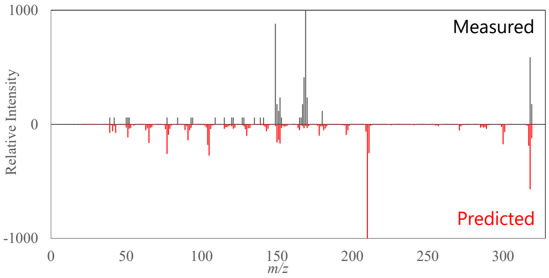
Rank:<br>544 (1707)<br>Formula:<br>$C_{11}H_{10}O_2$<br>Similarity:<br>0.75	Correct<br>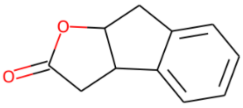<br>Top<br>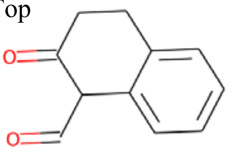	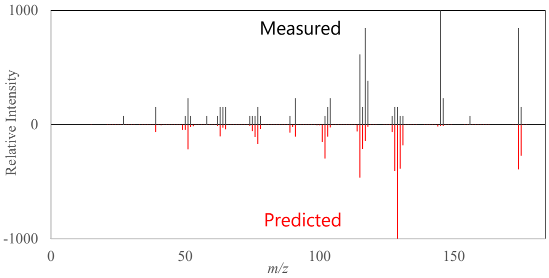
Rank:<br>26 (184)<br>Formula:<br>$C_{10}H_7N_5S$<br>Similarity:<br>0.66	Correct<br>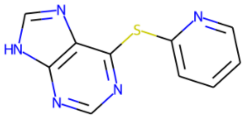<br>Top<br>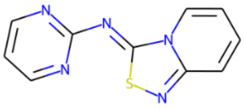	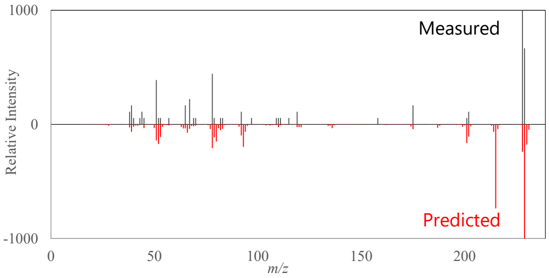
Rank:<br>130 (348)<br>Formula:<br>$C_{21}H_{15}N_3OS$<br>Similarity:<br>0.61	Correct<br>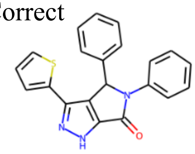<br>Top<br>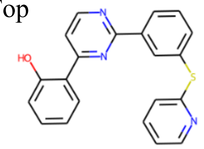	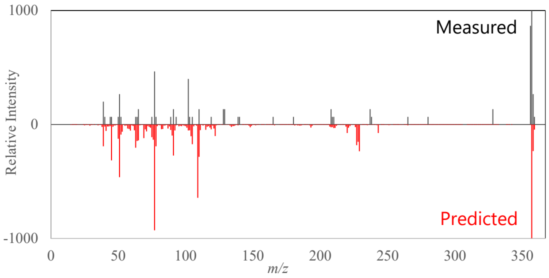
Rank:<br>25 (209)<br>Formula:<br>$C_{14}H_{12}F_3NO_4$<br>Similarity:<br>0.49	Correct<br>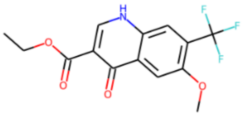<br>Top<br>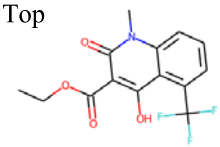	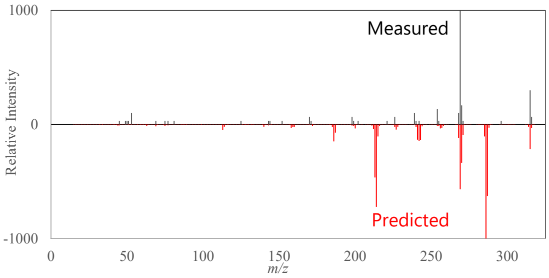

Table S5 continued

Rank:<br>39 (357)<br>Formula:<br>$C_{18}H_{11}NO_2$<br>Similarity:<br>0.78	Correct<br>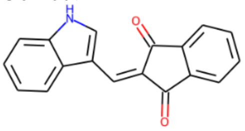<br>Top<br>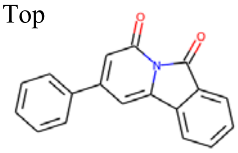	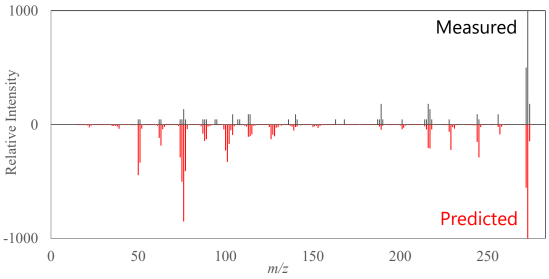
Rank:<br>155 (880)<br>Formula:<br>$C_{12}H_9ClN_4O$<br>Similarity:<br>0.34	Correct<br>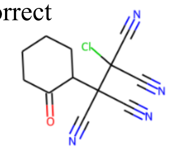<br>Top<br>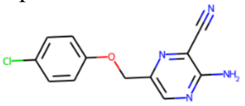	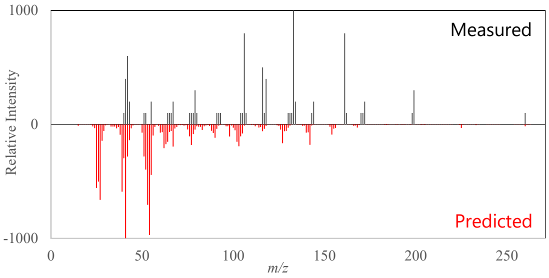
Rank:<br>69 (314)<br>Formula:<br>$C_{12}H_{10}$<br>Similarity:<br>0.84	Correct<br>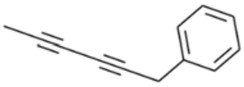<br>Top<br>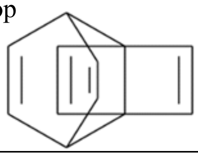	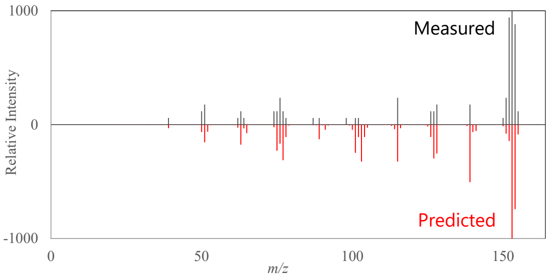
Rank:<br>889 (2290)<br>Formula:<br>$C_{20}H_{28}O_3$<br>Similarity:<br>0.55	Correct<br>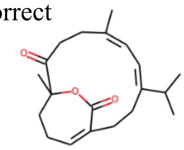<br>Top<br>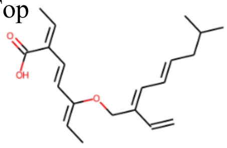	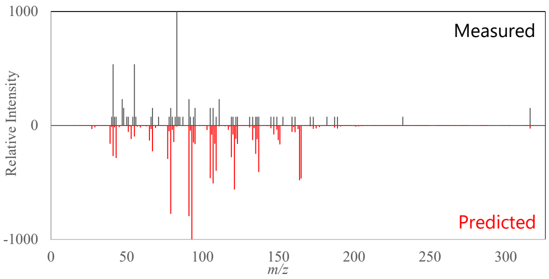
Rank:<br>32 (180)<br>Formula:<br>$C_7H_{11}IO_2$<br>Similarity:<br>0.59	Correct<br>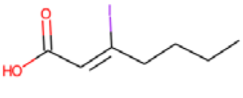<br>Top<br>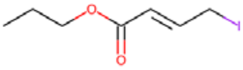	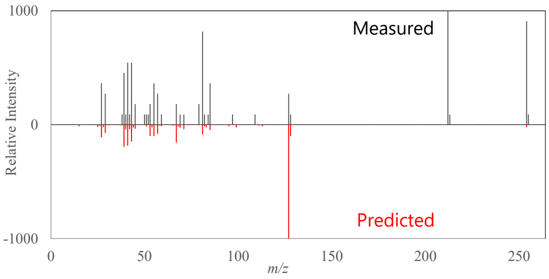

Table S6 Accuracy evaluation result of the pEI library search method for six compounds that were not registered in NIST 20

Compound Name	Structure	Similarity	Rank	Top 10 structures
Cafenstrole	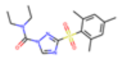	**0.741**	**3 (2933)**	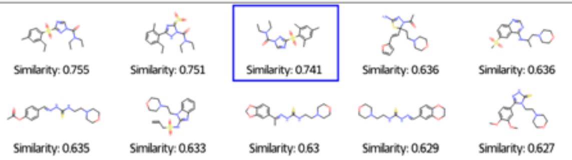
MCPA-thioethyl	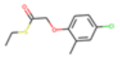	**0.735**	**1 (729)**	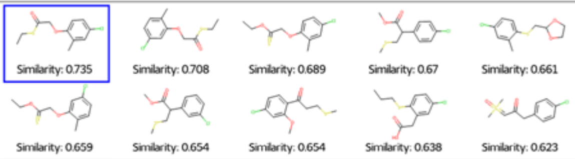
Propaphos	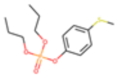	**0.802**	**1 (27)**	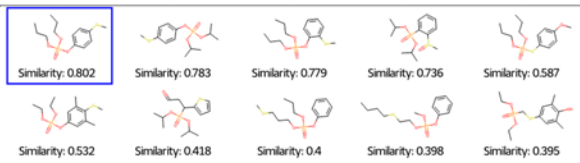
CNP-amino	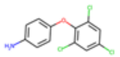	**0.710**	**14 (618)**	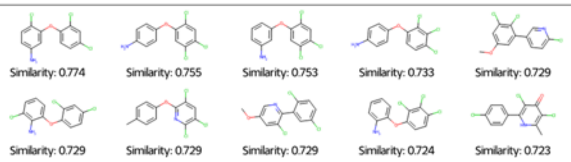
Butamifos oxon	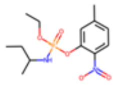	**0.675**	**1 (56)**	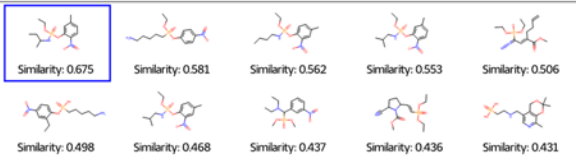
Isoxadifen-ethyl	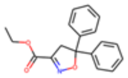	**0.586**	**22 (5348)**	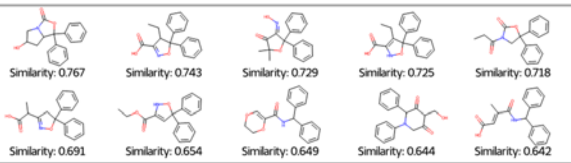
